# A Compound Heterozygous Mutation in *Calpain 1* Identifies a New Genetic Cause for Spinal Muscular Atrophy Type 4 (SMA4)

**DOI:** 10.3389/fgene.2021.801253

**Published:** 2022-01-19

**Authors:** G. Perez-Siles, M. Ellis, A. Ashe, B. Grosz, S. Vucic, M. C. Kiernan, K. A. Morris, S. W. Reddel, M. L. Kennerson

**Affiliations:** ^1^ Northcott Neuroscience Laboratory, ANZAC Research Institute, Sydney, NSW, Australia; ^2^ Sydney Medical School, University of Sydney, Sydney, NSW, Australia; ^3^ Brain and Mind Centre, The University of Sydney, Sydney, NSW, Australia; ^4^ Brain and Nerve Research Center, Concord Clinical School, University of Sydney, Sydney, NSW, Australia; ^5^ Department of Neurology, Royal Prince Alfred Hospital, Sydney, NSW, Australia; ^6^ Department of Neurology, Concord Repatriation General Hospital, Sydney, Sydney, NSW, Australia; ^7^ Molecular Medicine Laboratory, Concord Repatriation General Hospital, Sydney, NSW, Australia

**Keywords:** spinal muscular atrophy, calpain, patient fibroblasts, non-SMN1, β-catenin

## Abstract

Spinal Muscular Atrophy (SMA) is a heterogeneous group of neuromuscular diseases characterized by degeneration of anterior horn cells of the spinal cord, leading to muscular atrophy and weakness. Although the major cause of SMA is autosomal recessive exon deletions or loss-of-function mutations of *survival motor neuron 1* (*SMN1*) gene, next generation sequencing technologies are increasing the genetic heterogeneity of SMA. SMA type 4 (SMA4) is an adult onset, less severe form of SMA for which genetic and pathogenic causes remain elusive.Whole exome sequencing in a 30-year-old brother and sister with SMA4 identified a compound heterozygous mutation (p. G492R/p. F610C) in calpain-1 (*CAPN1*)*.* Mutations in *CAPN1* have been previously associated with cerebellar ataxia and hereditary spastic paraplegia. Using skin fibroblasts from a patient bearing the p. G492R/p. F610C mutation, we demonstrate reduced levels of *CAPN1* protein and protease activity. Functional characterization of the SMA4 fibroblasts revealed no changes in SMN protein levels and subcellular distribution. Additional cellular pathways associated with SMA remain unaffected in the patient fibroblasts, highlighting the tissue specificity of *CAPN1* dysfunction in SMA4 pathophysiology. This study provides genetic and functional evidence of *CAPN1* as a novel gene for the SMA4 phenotype and expands the phenotype of *CAPN1* mutation disorders.

## Introduction

Spinal muscular atrophies (SMAs) are a clinically and genetically heterogeneous group of disorders in which the selective loss of spinal cord motor neurons, progressive muscle denervation, and skeletal muscle atrophy are the main pathological components (Crawford and Pardo, 1996). *Survival of Motor Neuron 1* gene (*SMN1,* MIM#600354), located on chromosome 5q13.2 was initially identified as the childhood SMA causative gene (Lefebvre et al., 1995), soon after mapping this autosomal recessive disorder to chromosome 5q (Brzustowicz et al., 1990). The typical change is a deletion of at least exon 7 of *SMN1* on both alleles, with 2–5% of cases having compound deletion and inactivating mutation on each allele (Prior et al., 1993).

Although the majority of SMN protein is translated from the telomeric *SMN1* gene, expression of SMN in humans also relies on the *SMN2* gene (MIM#601627), an almost identical centromeric copy of *SMN1*. The *SMN2* copy has a C to T nucleotide substitution in exon 7 (Lorson et al., 1999). This single base change causes aberrant splicing and exclusion of exon 7 in *SMN2* (Monani et al., 1999) which produces 90% expression of a truncated, less stable, and dysfunctional SMNΔ7 protein, leaving only 10% functional full-length SMN (Chang et al., 2004). The severity of dysfunction in SMA is inversely proportional to the number of copies of *SMN2* and forms the basis for clinical classification of SMA subtypes (Lefebvre et al., 1997). Type 4 SMA (SMA4; MIM#271150) is the adult-onset form of SMA in which affected patients show 4 or more copies of *SMN2*. Patient diagnosis and initial symptoms usually present in the second or third decade of life starting with muscle weakness in the lower extremities. This muscle weakness progresses to affect independent walking and the upper limbs; in its mildest form walking can be achieved unaided and life expectancy remains unaffected (D'Amico et al., 2011).

Deletions and point mutations in *SMN/1* account for 96% of SMA patients, with 4% being unlinked to 5q13 (Wirth, 2000). The development of next generation sequencing technologies is increasing the number of genes associated with these non-5q SMA patients. These genes are involved in a variety of cellular and biological processes (Farrar and Kiernan, 2015) and research interest has focused on establishing a functional connection that links non-SMN-related and SMN-related spinal muscular atrophy (Soltic and Fuller, 2020). However, many of the other described causes of SMA are clinically dissimilar to SMN1 associated SMAs.

In this study, we present a family study of clinically typical phenotype SMA4 in which mutation analysis of the male proband and his affected sister was excluded for deletions and mutations in the *SMN1* gene. Whole exome sequencing of family members identified a compound heterozygous mutation (p. G492R/p. F610C) within the calpain-1 (*CAPN1*) gene. Calpains are a calcium-activated cysteine family of proteases involved in numerous cellular processes, including myogenesis, muscle remodelling, and synaptic function (Nath et al., 1996; Campbell and Davies, 2012; Briz and Baudry, 2017). Loss-of-function mutations in *CAPN1* are known to cause cerebellar ataxia (Wang et al., 2016) and hereditary spastic paraplegia (HSP) (Gan-Or et al., 2016) with the number of ataxia and/or pure HSP patients reporting *CAPN1* mutations rapidly increasing (Mereaux et al., 2021). Interestingly, recent investigations described the role of *CAPN1* regulating SMN (Wang et al., 2019) (de la Fuente et al., 2020) and a positive effect on SMA *in vitro* and *in vivo* models when treated with calpain inhibitors (de la Fuente et al., 2019). These findings suggest that both reduced and overactivated calpain can be associated with neurodegeneration and cause distinct clinical phenotypes. In the present work, using SMA4-derived skin fibroblasts bearing the p. G492R/p. F610C substitutions we demonstrate this compound heterozygous mutation reduces *CAPN1* protein levels and protease activity. Our findings provide for the first time genetic evidence for *CAPN1* as a novel non-5q-SMA causative gene.

## Materials and Methods

### Research Guidelines and Regulations

All research and cell culture procedures were conducted following written consent according to protocols approved by the Sydney Local Health District Human Ethics Review Committee, Concord Repatriation General Hospital, Sydney, Australia (reference number: HREC/11/CRGH/105). Informed consent for study participation was obtained from all patients and controls. All research was performed in accordance with relevant guidelines and regulations.

### Genetic Studies

Prior to this study the proband was tested for the mutations in the *SMN1* and *SMN2* genes. Whole exome sequencing was outsourced to Macrogen (South Korea) as previously described (Drew et al., 2015). Sequence reads were mapped to the GRCh37/hg19 assembly. The mutations were validated with Sanger sequencing using BigDye Terminator Cycle Sequencing protocols at the ACRF Facility, Garvan Institute of Medical Research (Australia). Data filtering was performed using multiple publicly available databases including gnomAD (https://gnomad.broadinstitute.org/) (Karczewski et al., 2020), 1,000 Genomes (http://www.1000genomes.org/), and Exome Variant Server (https://evs.gs.washington.edu/EVS/). Evolutionary conservation of the *CAPN1* p. G492 and p. F610 amino acid residues was assessed *in silico* using GERP (http://mendel.stanford.edu/sidowlab/downloads/gerp/index.html) (Davydov et al., 2010), phastCons (Siepel et al., 2005) and PhyloP (Pollard et al., 2010) (http://compgen.cshl.edu/phastweb/runtool.php). The predicted functional effect of the *CAPN1* p. G492R and p. F610C mutations was assessed separately *in silico* using Polyphen2 (http://genetics.bwh.harvard.edu/pph2/) (Adzhubei et al., 2010), PROVEAN (http://provean.jcvi.org/seq_submit.php) (Choi et al., 2012), SIFT (https://sift.bii.a-star.edu.sg/www/SIFT_seq_submit2.html) (Sim et al., 2012), MutationAssessor (http://mutationassessor.org/r3/) (Reva et al., 2007), and MutationTaster2 (http://www.mutationtaster.org/) (Schwarz et al., 2014). Pathogenicity was interpreted using the American College of Medical Genetics and Genomics (ACMG) and the Association for Molecular Pathology (AMP) guidelines (Richards et al., 2015).

### Subjects, Cell Culture Maintenance and Growing Conditions

Primary fibroblasts were cultured from a SMA4 patient and 3 neurologically normal skin biopsies and maintained with fibroblast cell culture medium (FDMEM): DMEM (Gibco, Life technologies) supplemented with 10% (v/v) fetal bovine serum (SAFC Biosciences), 1% (v/v) Penicillin Streptomycin (Gibco, Life technologies) and 1% (v/v) l-glutamine (Gibco, Life technologies) and maintained at 37°C in humidified air and 5% CO2.

### Preparation of Protein From Cell Lysates

Fibroblast cells were cultured until confluency for preparation of cell lysates for western blot analysis and determining *CAPN1* specific activity using RIPA buffer (150 mM NaCl, 1% (v/v) Triton X-100, 0.5% (w/v) sodium deoxycholate, 0.1% (w/v) SDS, 50 mM Tris pH8.0, 1x cOmplete™ EDTA-free protease inhibitor cocktail tablet). Lysis buffer additionally contained 5 mM ethylenebis (oxyethylenenitrilo)tetraacetic acid (EGTA) and added 2 mM CaCl_2_ when indicated. Concentration of protein in the cells lysates was determined using the Pierce BCA Protein Assay Kit (ThermoScientific).

### Western Blotting

Samples, SMA4 patient (proband II:2) and controls (*n* = 3), were prepared with 4x Laemmli sample buffer (BioRad) and 2-mercaptoethanol (Sigma) solution 9:1 (v/v). Cell lysates (15 μg) were size fractioned using mini-PROTEAN TGX Precast Gels (BioRad) and then transferred to an Immobilon®-P Polyvinylidene difluoride membrane (Sigma). Membranes were probed with the following primary antibodies: rabbit α-*CAPN1* (CST), 1:1,500; mouse α-SMN (BD Transduction Laboratories), 1:2000; rabbit α-*p*-Akt (CST), 1:1,000; mouse α-Pan-Akt (CST), 1:2000; rabbit α-LC3B-II (CST) 1:1,000; rabbit α-β-actin (CST) 1:2000; rabbit α-caspase-3 (Abcam) 1:4,000 and rabbit α-β-actin (CST) 1:2000. Secondary antibodies used in this study include goat α-rabbit IgG HRP (Sigma), 1:10,000 and goat α-mouse IgG HRP (abcam) 1:5,000. Immobilon™ Western Chemiluminescent HRP Substrate Reagent (Millipore) was added and protein bands were visualized using a ChemiDoc™ MP Imaging System (BioRad).

### Immunohistochemistry

48 h before the experiment, 1.5 × 10^4^ cells were plated onto optically clear bottom 96-well plates (CellCarrier-96, PerkinElmer). Cells were treated with 5 mM EGTA or 2 mM CaCl_2_ for 16 h before the experiment. Cells were washed once using PBS, fixed with 4% (v/v) paraformaldehyde for 12 min at room temperature (RT), permeabilized in phosphate-buffered saline (PBS) containing 0.3% (v/v) Triton X-100 and blocked in 5% (w/v) bovine serum albumin (BSA) for 60 min. Cells were incubated with the following primary antibodies overnight in 4°C: mouse α-SMN (BD Transduction Laboratories), 1:200; rabbit α-LC3B-II (Cell Signaling), 1:200. After washing three times with PBS, cells were incubated with Alexa Fluor secondary antibodies (Invitrogen) for 2 h at RT. Alexa Fluor 647 Phalloidin (ThermoFisher) was added to the secondary antibodies for staining of the fibroblasts’ membrane and segmentation purposes in the open-source software image analysis, *CellProfiler* 4.0.6 (https://cellprofiler.org/) using in-house pipelines. Nuclei were stained with 300 nM 4,6-diamidino-2- phenylindole (DAPI, Molecular Probes). Cells were visualized using a Leica SP8 confocal microscope equipped with a motorised stage for automated acquisition of images, that were acquired at ×20 magnification and ×2.5 digital zoom.

### TMRE Staining

48 h before the experiment, 1.5 × 10^4^ cells were plated onto optically clear bottom 96-well plates (CellCarrier-96, PerkinElmer). Cells were washed once using PBS and incubated with 1 μM TMRE (Thermofisher) in FDMEM for 45 min at 37°C. Cells were washed once using PBS and nuclei were stained with 1 μg/ml Hoechst in PBS for 10 min, followed by a final wash in PBS. 10 random images were taken using a Leica SP8 confocal microscope as indicated above. TMRE staining per cell was quantified using *CellProfiler* 4.0.6.

### Calpain Assay

The hydrolysis of the fluorogenic substrate Succ-LLVY-AMC (Abcam) by calpains in patient and control fibroblast lysates was performed in a reaction buffer containing 25 mM Tris-HCI, 145 mM NaCl (pH 7.4) and 0.5 mM EGTA in the absence or presence of calcium as previously described (Chelko et al., 2021) with some modifications. To specifically determine *CAPN1* activity, these experiments were performed in the presence of 1 µM PD151746 (C1I), a recently developed calpain inhibitor showing high specificity for *CAPN1* (calpain-1: Ki = 260 nM; calpain-2: Ki = 5.33 µM). The reaction was initiated by adding, 5 mM CaCl_2_ (3 mM free Ca^2+^) and the 100 μM Suc-Leu-Tyr-AMC enzyme to each sample containing 100 µg protein, continuing at 30°C for 45 min. The fluorescence of 7-amino-4- methylcoumarin (Ex 380 nm/Em 450 nm) was monitored every 30s using an EnSpire™ Multimode Plate Reader (PerkinElmer). The rate of hydrolysis (increase in fluorescence/second) was determined from the linear portion of the curve. *CAPN1* activity was estimated by subtracting the rate of hydrolysis in the presence of 1 µM C1I from the value obtained in the absence of calpain inhibitors. Free calcium concentrations were calculated using the website www.maxchelator.stanford.edu/CaEGTA-TS.htm.

### Molecular Modelling

The PyMOL program (Schrodinger) was used to localise the p. F610 and p. G492 residues into the ribbon structure diagram of the *Rattus norvegicus* *CAPN1* protein (PDB:1QXP). The impact of the variants on *CAPN1* protein stability was modelled and predicted using the PremPS online tool (https://lilab.jysw.suda.edu.cn/research/PremPS/). *In silico* modelling changes in protein-protein interaction between calpain2 and calpastatin (PDB:3BOW) were predicted using mCSM-PPI2 (http://biosig.unimelb.edu.au/mcsm_ppi2/).

### Statistical Analysis

For the statistical analysis, three independent experiments under the same conditions were performed and 2-way ANOVA followed by Tukey’s post hoc test used to assess the significance of the results. The data are expressed as mean ± SEM. The following statistical thresholds have been applied throughout the study: *p < 0.05; **p < 0.01; ***p < 0.001, ****p < 0.0001.

## Results

### Clinical and Electrodiagnostic Findings

**FIGURE 1:**
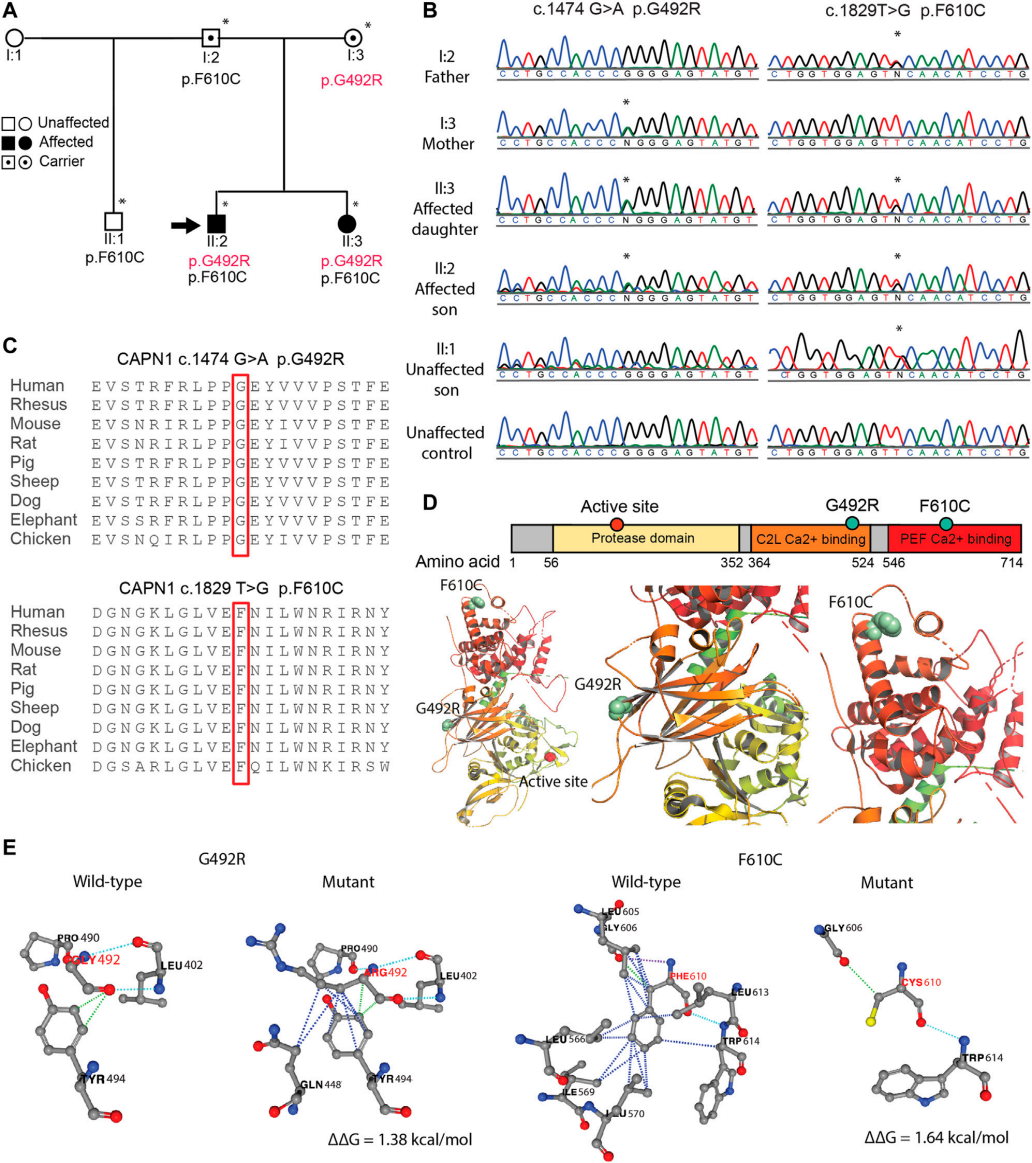
SMA4 causative p. G492R/p. F610C compound het mutation in *CAPN1*. **(A)** Pedigree of SMA4 family. Squares indicate male, circles indicate females. Solid symbols denote affected individuals and open symbols unaffected individuals. Carriers are represented with internal dots. Asterisk indicates individuals in which whole exomes sequencing was performed and the black arrow shows the proband. **(B)** Sanger sequencing confirms the nucleotide changes c.1474G > A (p.G492R) and c.1829T > G (p.F610C) in the two affected siblings (II:2 and II:3). **(C)** The substituted amino acid residues (p.G492 and p. F610) are located within highly conserved functional domains of the *CAPN1* protein. **(D)** *CAPN1* secondary structure displaying the protease domain (yellow) and the C2 domain-like (C2L, orange) and penta-EF-hand (PEF, red) Ca^2+^ binding domains bearing the p. G492R and p. F610C mutations respectively. Affected residues located in the 3D ribbon structure of *CAPN1* using PyMOL and the *Rattus norvegicus* *CAPN1* crystalized structure (PDB:1QXP). **(E)** The impact of the p. G492R and p. F610C missense mutations in the protein stability (∆∆G) of *CAPN1* predicted using the PremPS online tool. For clarity, the ribbon was hidden and only the non-covalent interactions affected by the substitutions are displayed. Dotted lines represent hydrophobic (blue), polar (light blue), hydrogen bonds (violet) and Van der Waals (green) interactions in the wild-type and mutant structures. Positive ∆∆G predicts a reduction in the stability of the resulted protein.

This research examines a family study of SMA4 in which the male proband presented in 2011 aged 36, onset of symptoms was uncertain but older than 2 years. The presenting symptoms were of increasing painless weakness, without sensory loss, propensity to falls, plus cramps in upper and lower limbs following usage. The affected family members have now been followed for 10 years. The clinical findings on presentation were of normal cranial nerves, symmetrical proximal > distal limb weakness, with disproportionate weakness of triceps and quadriceps. There was shoulder girdle, latissimus dorsi, triceps and quadriceps wasting and a waddling gait. Strength in the fingers and toes was normal without muscle hypertrophy. MRC scores are presented in Supplementary Table S1. Fasciculations were visible in proximal upper and lower limbs. There was no action nor rest tremor. The reflexes were depressed at triceps, absent knee jerks, other limb reflexes present, normal coordination, sensation and sphincter function. The proband’s full sister was first seen aged 34, but reported difficulty getting off the floor from age 21. Clinical findings were essentially the same. The proband’s half-brother, parents and son were clinically normal (Figure 1A).

Neurophysiology showed normal nerve conduction study findings with normal compound muscle action potential amplitudes, distal motor latencies and motor nerve conduction velocities of the median, ulnar, tibial and peroneal nerves, and normal sensory studies of the median, ulnar and sural nerves. Electromyography in 2011 of the proband of a limited selection of limb muscles consistently showed some active features on insertion at rest, and a chronic neurogenic reinnervation pattern on activation with increased motor unit amplitudes and polyphasia, more proximally than in the more distal tibialis anterior. A high isolated firing rate (early recruitment) was observed on activation with decreased interference pattern at full activation. Details are provided in Supplementary Table S2. Quantitative EMG was not performed and electrophysiology has not been repeated in recent years.

Creatine kinase level was 466 U/ml (normal range 22–198 U/ml). Deltoid muscle biopsy showed grouped atrophic fibres of both fibre types consistent with chronic neurogenic changes. MRI spine and a lumbar puncture were non informative or normal.

### Genetic Analysis

Clinical testing of the *SMN1* and *SMN2* in the proband excluded mutations in the *SMN1* gene. Subsequent whole exome sequencing of the proband and his sister identified a compound heterozygous mutation involving two rare missense variants in the *CAPN1* gene. The variants were located in exon 13 and exon 18 (NM_001198868): c.1474G > A p. G492R [chr11:64,974,054 (hg19)] and c.1829T > G p. F610C [chr11:64,977,354 (hg19] respectively. Family segregation analysis showed the unaffected father and mother were carriers of the p. F610C and p. G492R mutation respectively. The unaffected half-brother of the proband was a carrier of p. F610C (Figure 1A). The mutations were confirmed by Sanger sequencing for all individuals (Figure 1B). Assuming linkage equilibrium, the probability of two SNPs occurring together in an individual can be calculated by multiplying the minor allele frequencies (MAF) of the SNPs (Slatkin, 2008). The reported MAF of c.1474G > A (rs17883283) is 0.001 and the reported MAF of c.1829T > G (rs200876514) is 0.0003, and therefore the possibility of them co-occurring in an individual is 0.00003% (3/10, 000, 000 individuals). Furthermore, the probability of two affected siblings inheriting c.1474G > A and c.1829T > G from their carrier mother and father respectively is 6.25% (1/16). This statistical unlikelihood of the results described here provides strong genetic evidence for the involvement of compound heterozygous *CAPN1* mutations in SMA4.

The amino acid residues p. G492 and p. F610 are located within highly conserved regions of the *CAPN1* protein (Figure 1C), and evolutionary constraint of these residues was further supported by *in silico* analysis (Supplementary Table S1). Additional computational tools further predicted a damaging effect of p. G492R and p. F610C on *CAPN1* function (Supplementary Table S3). Although most HSP-causative missense mutations are located in the protease domain of *CAPN1* (Lai et al., 2020), they can be found across the 3 functional domains of the protein. The p. G492 and p. F610 amino acid residues are in the C2-like domain and the penta-EF-hand domain (PEF) respectively, far from the *CAPN1* active site (p.C115), and not located near each other in the 3D structure of the protein (Figure 1D). *In silico* modelling the impact of the p. G492R and p. F610C mutations using the PremPS online tool predicts a change in the free energy of folding (∆∆G) of +1.38 kcal/mol and +1.64 kcal/mol respectively, suggesting pathogenicity of these substitutions through decreased stability of *CAPN1* (Figure 1E). According to the variant pathogenicity guidelines determined by ACMG-AMP, both the p. G492R and p. F610C variant can be separately classified as likely pathogenic (PM1, PM2, PP2, PP3).

### SMA4 Patient Fibroblasts Show Reduced *CAPN1* Protein Levels and *CAPN1* Activity

**FIGURE 2:**
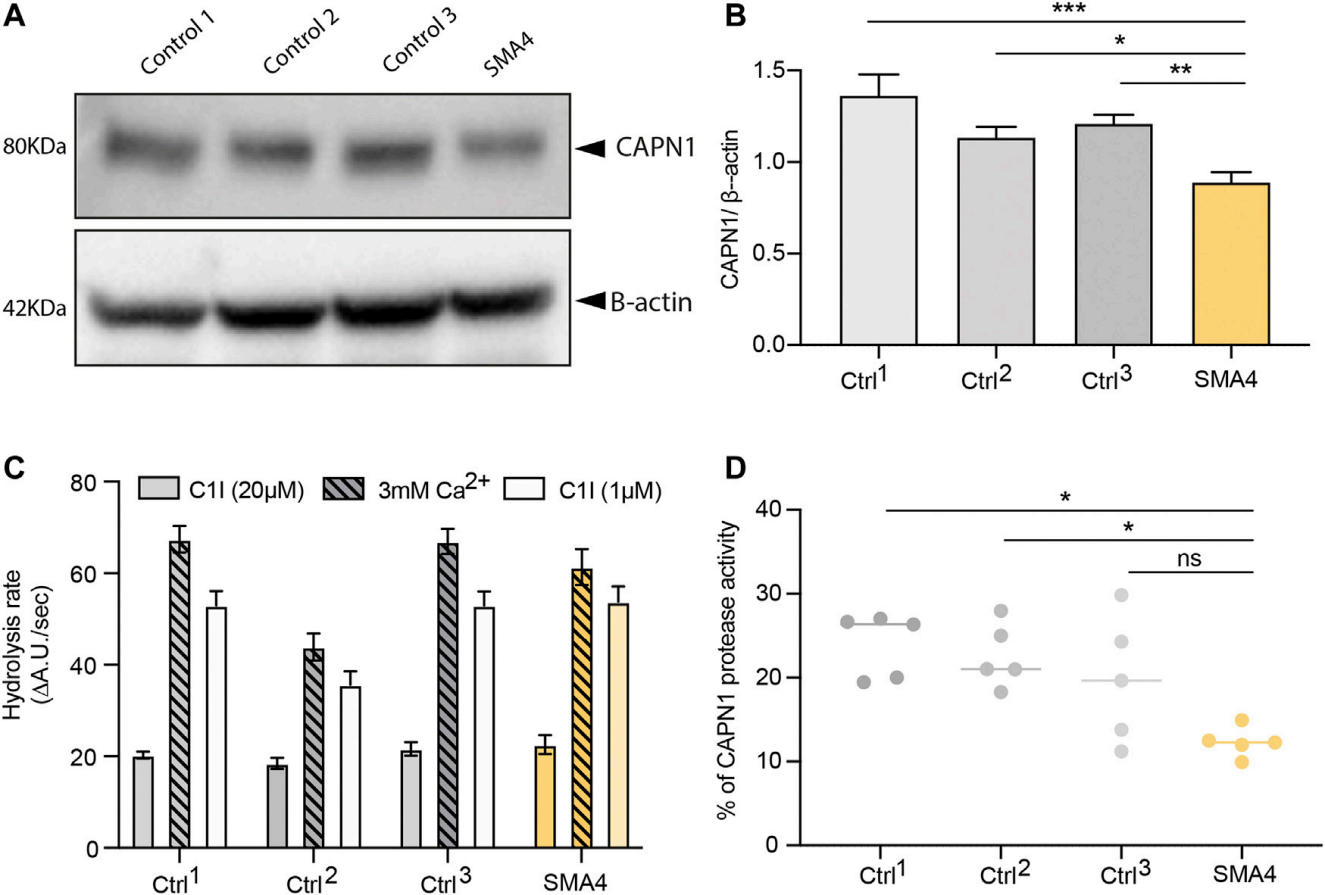
Consequences of p. G492R/p. F610C compound heterozygous mutation on *CAPN1* protein levels and protease activity. **(A)** Levels of *CAPN1* protein determined by western blot analysis in lysates from a SMA4 patient (proband II:2) compared to controls fibroblasts (*n* = 3). β-actin was used as a loading control in these experiments. **(B)** Data in bar graphs are represented as mean ± SEM from 3 independent experiments. **(C)** Protease activity determined from 100 μg protein lysates in the presence of 0.5 mM EGTA and 3 mM CaCl_2_ (Ca^2+^)_,_ with and without the specific calpain-1 inhibitor (C1I) at saturating (20 µM) and at half-maximal inhibitory concentration (1 µM). Data in bar graphs represents the mean ± SEM of the hydrolysis rate of 100 μM Succ-LLVY-AMC substrate determined from the linear portion of the curve measured every 30 s for 45 min. **(D)** Calpain-1 activity from 5 independent experiments. Data is represented as % of Succ-LLVY-AMC hydrolysis in the assay that can be attributed to *CAPN1*, calculated by dividing the hydrolysis rate in the presence of 3 mM free Ca^2+^ by the value obtained in the presence of 1 µM C1I. *p* values were obtained from a 2-way ANOVA test (*p < 0.05; **p < 0.005; ***p < 0.0005).

Mutations in *CAPN1* causing HSP lead to a reduction of *CAPN1* protein expression and are associated with diminished calpain protease activity in patient-derived cells (Wang et al., 2016). Recent research however associates overactivated calpain-1 in the pathogenesis of SMA, suggesting the utility of calpain inhibition in SMA therapy (de la Fuente et al., 2019; de la Fuente et al., 2020). To investigate the effect the p. G492R/p. F610C compound heterozygous mutation has on *CAPN1*, skin fibroblasts from the SMA4 patient (II:2) and 3 gender/age matched neurologically normal controls were cultured. Protein levels of *CAPN1* in fibroblasts lysates were examined by western blot (Figure 2A) and showed a reduction in *CAPN1* expression in the SMA4-derived cells when compared to the three control lines. Quantification of these bands (Figure 2B) demonstrated the patient cells have 35% less calpain-1 than control fibroblasts (x̅_SMA4_ = 0.88 versus x̅_Ctrls_ = 1.23 densitometry units). Calpain activity in fibroblasts lysates was determined measuring the hydrolysis rate of the fluorescent substrate Succ-LLVY-AMC in the presence of 3 mM free Ca^2+^. To specifically assess *CAPN1* activity, these experiments were performed in the presence of saturating (20 µM) and low concentration (1 µM) PD151746 (C1I), a calpain inhibitor showing high specificity for *CAPN1* (Figure 2C). Our experiments showed the SMA4 patient cells have reduced *CAPN1*-specific protease activity (% of Succ-LLVY-AMC hydrolysis rate in the assay that can be attributed to *CAPN1*, calculated by dividing the hydrolysis rate in the presence of 3 mM free Ca^2+^ by the value obtained in the presence of 1 µM C1I) when compared to all control lines (x̅_SMA4_ = 12% versus x̅_Ctrls_ = 22% of *CAPN1*-specific protease activity, Figure 2D).

### SMA4 Patient Fibroblasts Show No Changes in SMN1 Protein Levels or SMN1 Subcellular Distribution

**FIGURE 3:**
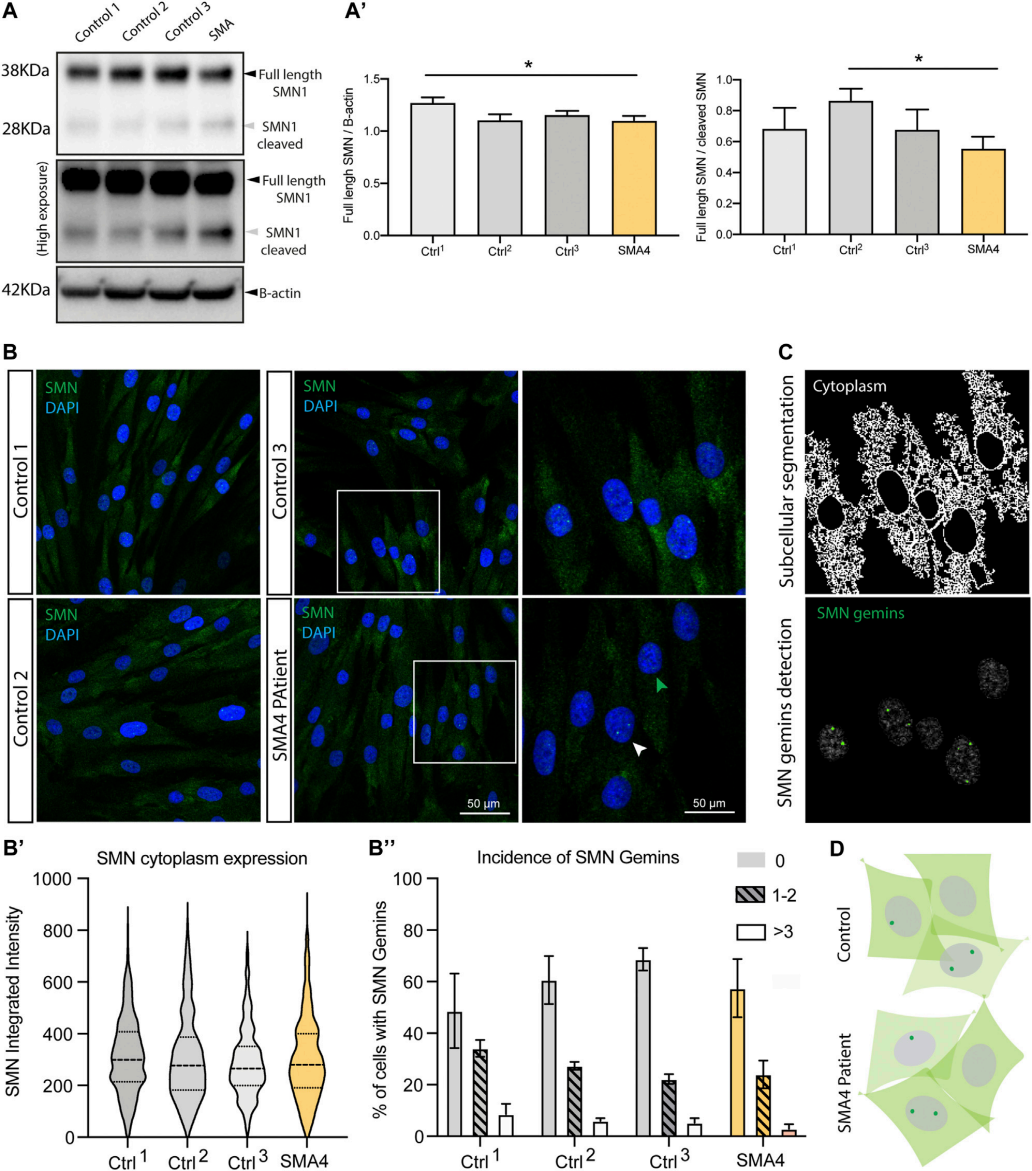
SMN protein levels subcellular distribution in SMA4 patient fibroblasts show no changes in SMN1 protein levels or SMN1 subcellular distribution. **(A)** Levels of SMN protein determined by western blot analysis in lysates from a SMA4 patient and control fibroblasts. β-actin was used as a loading control in these experiments. High exposition of the membrane blots allow visualization of both the full length (FL) SMN (38 KDa) and the cleaved SMN fragment (28 KDa). **(A)** The amount of FL SMN and FL SMN/cleaved SMN was determined and shown in bar graphs as the mean ± SEM for each cell line. **(B)** Immunofluorescence analysis in control and SMA4-derived fibroblasts detects SMN (green) distribution in cytoplasmic and within the nuclei (blue) localising to subnuclear structures (gemins). Boxed areas are enlarged to allow visualization of cells showing no gemins (green arrowhead) and nuclei with SMN-gemins (white arrowhead). **(B)** Quantification of the SMN cytoplasmic expression (integrated intensity) within each cell. Violin plot shows the full distribution of all data points acquired (*n* > 250 cells). **(B)** Incidence of SMN-gemins shown in bar graphs (as the mean ± SEM from 3 separate experiments for each cell line) and distributed in 3 separated groups, including cells showing no gemins (no pattern), cells showing 1 or 2 SMN-gemins (lined pattern) and nuclei in which 3 or more SMN gemins were detected (clear bar). **(C)** Representative example of the in-house developed *CellProfiler* pipeline to allow segmentation of the cytoplasm and nuclear gemins in the immunofluorescence experiments. **(D)** Representative cartoon showing subcellular expression of SMN in skin fibroblasts highlighting a similar distribution in control and SMA4-derived cells.

In approximately 4% of SMA patients, there is not a direct genetic causative link with *SMN1* (Wirth, 2000). Increasing evidence suggest however there is a functional association between these additional genes and SMN-related spinal muscular atrophy (Soltic and Fuller, 2020). SMN is a known proteolytic target of endogenous calpains (Walker et al., 2008) (Fuentes et al., 2010) and recent investigations demonstrate *CAPN1* regulate SMN levels *in vitro* (Wang et al., 2019) and *in vivo* (de la Fuente et al., 2020). To determine if reduced *CAPN1* protein and activity shown in the SMA4 patient cells (Figure 2) affects the intracellular levels and/or the subcellular distribution of SMN, western blot analysis and immunofluorescence experiments were performed (Figure 3). Cleavage of full length (FL) SMN (38 KDa) by calpains produces an N-terminal 28 KDa fragment distinctly detectable by immunoblotting. Protein lysates from the SMA4 patient and three controls were blotted using an anti-SMN antibody. Western blot analysis shows there is not a significant change in the levels of the FL SMN and/or cleaved 28 KDa fragment between affected and control lines (Figure 3A). Quantification of these bands (FL and FL/cleaved SMN) confirms the small variations observed don’t reached statistically significant differences across the three control lines utilised in this study (FL: x̅_SMA4_ = 1.09 versus x̅_Ctrls_ = 1.17 densitometry units; FL/cleaved SMN: x̅_SMA4_ = 0.55 versus x̅_Ctrls_ = 0.74 densitometry units; Figure 3A’).

SMN is expressed both in the cytoplasm and in the nuclei, where it localises to subnuclear bodies called gems (Liu et al., 1997). To assess whether the p. G492R/p.F610C compound heterozygous mutation has any effect on the intracellular localisation of SMN, immunofluorescence staining was performed (Figure 3B) and images segmented into nuclear and cytoplasmic compartments to allow the precise subcellular quantification of SMN (Figure 3C). Levels of cytoplasmic SMN were comparable between SMA4 fibroblasts and control cells and no changes were detected in the integrated density quantified in the cytoplasmic compartment (cytoplasmic SMN: x̅_SMA4_ = 303.5 versus x̅_Ctrls_ = 302.27, SD_Ctrls_ = 12.92 integrated intensity; Figure 3B’). The appearance of SMN nuclei gemins was classified in three categories that included nuclei showing no gems, nuclei showing either 1 or 2, or cells displaying 3 or more SMN gemins. Detailed quantification following these criteria confirmed the visual observation that no changes are detected in the SMA4-derived cells when compared to control fibroblasts (0 gems: x̅_SMA4_ = 57.48% versus x̅_Ctrls_ = 59.33%; 1–2 gems: x̅_SMA4_ = 24.00% versus x̅_Ctrls_ = 27.87%; >3 gems: x̅_SMA4_ = 2.91% versus x̅_Ctrls_ = 4.59% of all cells; Figure 3B’’).

### Interrogating Intracellular Pathways Affected in Spinal Muscular Atrophy in SMA4-Derived Fibroblasts

Cellular and molecular pathways leading to motor neuron degeneration in SMN-related and non-5q SMAs are not yet fully understood. To evaluate the occurrence of intracellular pathways that might be triggered by decreased *CAPN1* activity (Figure 2D) and had been previously associated with *in vitro* models of SMA, we investigated the incidence of autophagy mitochondrial function and apoptosis in SMA4 fibroblasts.

**FIGURE 4:**
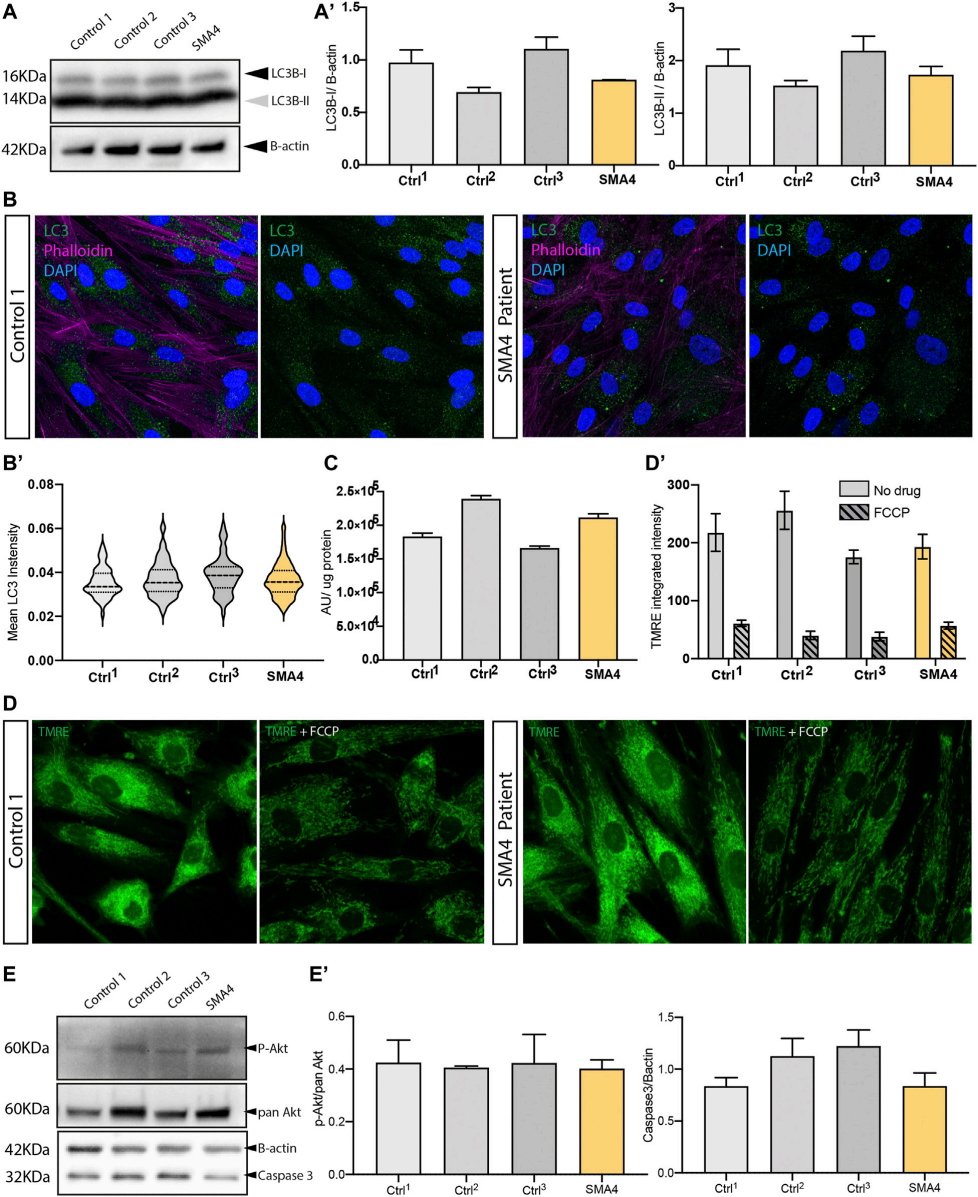
Effect of the *CAPN1* compound heterozygous mutation on intracellular pathways affected in spinal muscular atrophy. **(A)** Protein levels of the autophagy marker LC3B (LC3B-II, 14 KDa and LC3B-I, 16 KDa) determined by western blot analysis in lysates from SMA4 patient (proband II:2) compared to controls fibroblasts. β-actin was used as a loading control in these experiments. **(A)** The expression of LC3B type I (LC3B-I) and LC3B type II (LC3B-II) was determined and shown in bar graphs as the mean ± SEM from 3 independent experiments. **(B)** Autophagy occurrence visualised by confocal microscopy using an anti-LC3B antibody (green) in cells stained with phalloidin (violet). **(B)**. Quantification of the LC3B cytoplasmic expression within each cell. Violin plot shows the full distribution of all data points acquired (*n* > 250 cells). **(C)** ATP production was measured using the ATPlite assay kit. Arbitrary luminescence units (ALU) are shown for each experimental group from data obtained for 3 independent experiments. **(D)** Mitochondria membrane potential was assessed in live cells using 1 μM TMRE (green). 1 μM FCCP (carbonyl cyanide 4-(trifluoromethoxy) phenylhydrazone) was used as a positive control to uncouple mitochondrial oxidative phosphorylation. **(D)**. Quantification of the TMRE stanning (integrated intensity) for untreated (solid bars) and FCCP treated (lined pattern) cells was calculated for 10 random images and represented using bar graphs as the mean ± SEM from 3 separate experiments for each cell line. **(E)** Protein levels of the PI3K-Akt neuronal survival pathway protein *p*-Akt (relative to total pan Akt) and the proapoptotic caspase-3 determined by western blot analysis. β-actin was used as a loading control in these experiments. **(E)** The expression of *p*-Akt and caspase-3 was determined and shown in bar graphs as the mean ± SEM from 3 independent experiments.

Changes in the autophagosome formation and in the autophagy flux have been reported in SMA (Garcera et al., 2013) (Piras et al., 2017) and modulation of endogenous calpain associated with increased SMN availability through regulation of autophagy (Periyakaruppiah et al., 2016). To investigate autophagy in SMA4-derived cells we determined the expression of the autophagy marker LC3B by westernblot and immunofluorescence analysis (Figures 4A,B). Westernblot analysis indicated that the amount of LC3B-I and LC3B-II in the patient cells was within the normal range shown in the control fibroblasts (LC3B-I: x̅_SMA4_ = 0.81 versus x̅_Ctrls_ = 0.93, SD_Ctrls_ = 0.21; LC3B-II: x̅_SMA4_ = 1.73 versus x̅_Ctrls_ = 1.88, SD_Ctrls_ = 0.33 densitometry units Figure 4A’). Accordingly, quantification of the immunofluorescent staining shows no changes in the levels of cytosolic LC3B positive puncta between the SMA4 patient fibroblasts and controls (Figure 4B). Although recent data indicates a tissue-specific regulation in SMA of the autophagy process (Sansa et al., 2021b), our data suggest that the *CAPN1* compound heterozygous mutation doesn’t impact the autophagy process in SMA4-derived cells.

Mitochondrial dysfunction has been extensively associated with SMA pathophysiology and it is evident across the clinical spectrum of SMA [reviewed at (James et al., 2021)]. Interestingly, relocation of *CAPN1* into the mitochondria disrupts ATP synthase-α protein (ATP5A1) and ATP synthase activity (Ni et al., 2016) and recent research describes *CAPN1*-induced cleavage of mitochondrial-bound apoptosis-inducing factor (AIF) (Chelko et al., 2021). In this study we utilised the production of cellular ATP (Figure 4C), which main production source is the oxidative phosphorylation at the mitochondria, and the mitochondrial membrane potential marker TMRE (Figure 4D), that reflects the functional status and viability of mitochondria, as indicators of the mitochondrial health in SMA4-derived fibroblasts. Our data indicates a good correlation between ATP levels and membrane potential within each cell line (Figure 4C and Figure 4D’). These parameters in the SMA4 fibroblasts are within the normal range captured by the neurologically normal control cells (ATP levels: x̅_SMA4_ = 2.11 × 10^5^ versus x̅_Ctrls_ = 1.95 × 10^5^, SD_Ctrls_ = 0.39 × 10^5^ luminescence arbitrary units; TMRE: x̅_SMA4_ = 193.3 versus x̅_Ctrls_ = 215.77, SD_Ctrls_ = 41.54 integrated intensity) suggesting the *CAPN1* mutation does not impair mitochondrial function in our model.

The antiapoptotic role of SMN (Anderton et al., 2013) and the activation of apoptotic processes through the PI3K-Akt neuronal survival pathway has been stablished in SMA (Sansa et al., 2021a) and has been associated with *CAPN1* loss of function in cerebellar ataxia (Wang et al., 2016). We interrogated this pathway in SMA4 fibroblasts by westernblot analysis using anti phospho-Akt Ser473 (pAkt) and anti caspase-3 antibodies. Our experiments showed no statistically significant difference in the levels of pAkt when normalised against the total levels of Akt (pan Akt) or in the levels of caspase-3 in the SMA4 cells when compared to control fibroblasts (pAkt/pan Akt: x̅_SMA4_ = 0.40 versus x̅_Ctrls_ = 0.42, SD_Ctrls_ = 0.01; caspase-3: x̅_SMA4_ = 0.84 versus x̅_Ctrls_ = 1.06, SD_Ctrls_ = 0.20 densitometry units). This data suggests that the p. G492R/p. F610C compound heterozygous mutation does not induce apoptosis through the absence of the pro-survival Akt pathway.

### β-catenin as a Possible Molecular Crosstalk Between *CAPN1*, SMN and Non-5q SMA Genes

**FIGURE 5:**
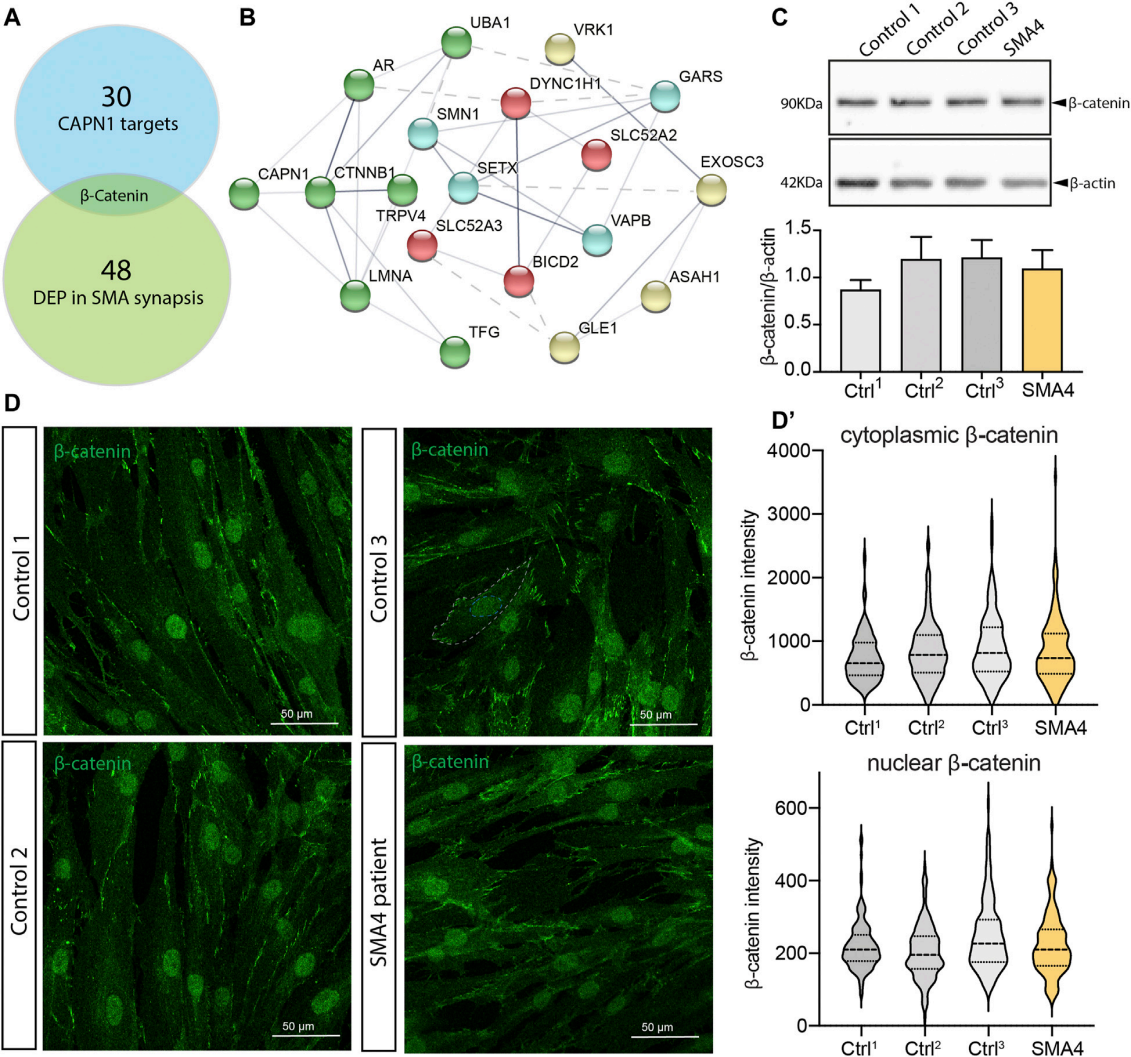
β-catenin is not altered in SMA4 patient fibroblasts. **(A)** β-catenin is the only differentially expressed protein in SMA synapses that is a *CAPN1* known protease target. **(B)** Functional protein association network of all reported SMA genes and *CTNNB1* using STRING. The thickness of the network edges indicates the strength of data support. Network clusters are differentiated by colour and their connection represented by a dotted line. **(C)** Protein levels of β-catenin determined by western blot analysis in lysates from a SMA4 patient compared to controls fibroblasts. β-actin was used as a loading control in these experiments. The expression of β-catenin is shown in bar graphs as the mean ± SEM from 3 independent experiments. **(D)** Immunofluorescence analysis in control and SMA4-derived fibroblasts detects β-catenin (green) distribution in cytoplasmic and within the nuclei **(D)**. Quantification of the β-catenin cytoplasmic expression and nuclear (integrated intensity) within each cell. Violin plot shows the full distribution of all data points acquired (*n* > 250 cells).

Multiomics studies are providing new insights into the pathomechanisms underlying SMA and identifying new molecular targets and potential therapeutic options (Meijboom et al., 2021). Within the set of proteins that appear differentially expressed in the synapse and spinal cords from mouse SMA models, β-catenin shows a 400% upregulation that is confirmed in muscle biopsies from SMA patients. Importantly, the list of proteins differentially expressed in SMA is significantly enriched in β-catenin targets, suggesting β-catenin may present a major feature of neuromuscular pathology in SMA (Wishart et al., 2014). When we compared the data set of proteins differentially expressed in these SMA models to a published list of direct targets of the protease activity of *CAPN1* (Shinkai-Ouchi et al., 2016), β-catenin is the only common protein we identified (Figure 5A). Pathway analysis shows a strong link between all SMA genes and *CTNNB1*, the gene that encodes β-catenin, with *CAPN1* clustering with *CTNNB, LMNA, AR, UBA1* and *TFG* (Figure 5B, green spheres) and *GARS* and *SMN1* being at the edge of a second cluster (Figure 5B, blue spheres) connected with the first one by *UBA1* (shown by the dotted line). Reduced *CAPN1* activity shown in SMA4 fibroblasts (Figure 5D) may lead to accumulation of β-catenin in the patient cells. Western blot analysis however indicates the levels of β-catenin in the SMA4 fibroblasts are within the range shown by the three control lines used in this study (x̅_SMA4_ = 1.09 versus x̅_Ctrls_ = 1.09, SD_Ctrls_ = 0.19 densitometry units; Figure 5C). In cells, β-catenin is mostly expressed in the nuclei where it regulates gene expression (Behrens et al., 1996) (Molenaar et al., 1996) and at the plasma membrane associated with E-cadherin regulating cell adhesion (Yap et al., 1997). Immunofluorescence staining confirms localisation of β-catenin in these locations (Figure 5D) and quantification of nuclear and cytoplasmic expression indicates β-catenin levels are comparable between SMA4 fibroblasts and control cells in these compartments (Nuclear β-catenin: x̅_SMA4_ = 225.50 versus x̅_Ctrls_ = 224.60, SD_Ctrls_ = 22.34; cytoplasmic β-catenin: x̅_SMA4_ = 862.30 versus x̅_Ctrls_ = 834.83, SD_Ctrls_ = 80.28 integrated intensity; Figure 5D’). The role of the Wnt/β-catenin signalling as a common molecular pathway in which SMN and non-5q SMAs may converge has recently been suggested (Soltic and Fuller, 2020). Our experiments using skin fibroblasts however suggests this association may be tissue-specific and further experiments using patient-derived motor neurons may provide further evidence of the functional association between *CAPN1*, β-catenin and degeneration of motor neurons in SMA4 patients.

## Discussion

Over 96% of SMA patients receive a genetic diagnosis based on loss of function deletions or mutations on the *SMN1* gene. These cases represent the more severe SMA types 1, 2 and generally type 3 cases, in which a prompt and early intervention is needed to minimise the unreversible and devastating clinical consequences following motor neuron loss. Research interest has focused accordingly in identifying venues to increase cellular availability of SMN protein. SMA research has pioneered gene-targeted therapy with currently 3 SMN-targeted therapies (*SMN2* splicing modifiers and *SMN1* gene replacement) approved by the FDA/EMA and a larger number are in clinical development (Chaytow et al., 2021).

Approximately 4% of SMA patients are not genetically linked to 5q13 and, although these cases belong to the less severe SMA types 3 and 4 or in many cases have a somewhat different phenotype, there is an urgent need for developing SMN-independent therapies to address motor neuron degeneration in these patients. In this regard, next generation sequencing technologies are increasing the number of genes associated with these non-5q SMAs and are therefore providing new pieces of evidence to identify a functional connection among the cellular and biological processes affected by these mutations. In this work we identified *CAPN1* as a new genetic cause for SMA type 4. The two affected siblings bear a compound heterozygous mutation (c.1474G > A and c.1829T > G) inherited from their unaffected father and mother, respectively. Importantly, the proband’s half-brother bearing the c.1474G > A substitution is clinically normal, providing strong genetic evidence for the involvement of *CAPN1* mutations in the current absence of additional SMA patients/families bearing mutations in this gene.

Calpains are evolutionarily conserved and widely expressed Ca^2+^-activated cysteine proteases and increasing evidence support their role in neuronal remodelling and neurodegeneration. The activity of calpains is tightly regulated and both reduced protease activity and abnormal activation can have deleterious effects, leading to impaired and promiscuous cleavage of various targets, respectively (Metwally et al., 2021). In this regard, previously identified *CAPN1* mutations are a known genetic cause for cerebellar ataxia (Wang et al., 2016) and hereditary spastic paraplegia (HSP) (Gan-Or et al., 2016). In these cases, functional evidence points to *CAPN1* loss of function as the underlying pathomechanism. Interestingly, until our study there had not been genetic evidence linking calpain function with SMA development, although recent investigations have described the role of *CAPN1* regulating SMN protein (Wang et al., 2019) (de la Fuente et al., 2020), suggesting the use of calpain inhibitors as a therapeutic strategy for SMA treatment (de la Fuente et al., 2019).

**FIGURE 6:**
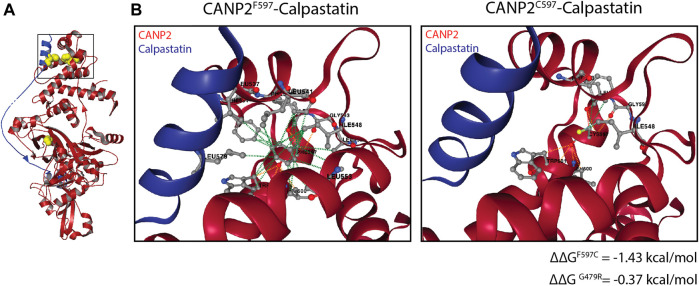
Impact of the p. G492R and p. F610C missense mutations in the calpain-calpastatin interaction. **(A)** Overall view of calpastatin-bound calpain-2 (PDB: 3bow) showing the calpain molecule in red and the calpastatin polypeptide in blue. G492 and F610 residues (G479 and F597 in calpain 2) and L578 (calpastatin) are shown as yellow spheres. **(B)** Non-covalent interactions maintaining the calpain-calpastatin complex, showing hydrophobic (green) and polar (orange) between the F597 (calpain) and L578 (calpastatin) residues.

We therefore sought to determine whether the compound heterozygous mutation in *CAPN1* leads to loss of function of the resulting protein or to increased protease activity. Our experiments demonstrate a 35% reduction in the *CAPN1* protein levels in the SMA4-derived fibroblasts. Accordingly, *CAPN1* activity in the patient cells accounts for 12% of total Ca^2+^ activated protease activity versus a 22% reported for the control lines. While this suggests the p. G492R/p. F610C mutation reduces *CAPN1* activity, this may only be a direct consequence of the reduced availability of CAPN1 in the SMA4 cells, associated with the reduced stability of the mutant *CAPN1* predicted by *in silico* modelling (Figure 1F). Importantly, our current experimental approach can’t exclude the hypothesis of increased protease activity of the mutant *CAPN1* in motor neurons from the SMA patient. Tissue specificity of calpain activity has recently been demonstrated (de la Fuente et al., 2020). De la Fuente *et al* (2020) showed opposite calpain-1 activity profiles in SMA fibroblasts (downregulation) and SMA-derived iPSC motor neurons (increased protease activity), although calpain-1 protein levels were reduced in both tissues. Damaging calpain overactivation has been associated with the pathogenic reduction of the natural endogenous calpain inhibitor calpastatin *in vivo* models of Alzheimer’s disease (Rao et al., 2014), amyotrophic lateral sclerosis (Rao et al., 2016) and Huntington’s disease (Hu et al., 2021). Although merely speculative, the 3D structure of *Rattus norvegicus* calpain-2 (CANP2) in association with calpastatin (Hanna et al., 2008) provides a plausible hypothesis for the potential impact the p. G492R/p. F610C compound heterozygous mutation may have on *CAPN1* regulation by its endogenous inhibitor. Calpastatin binds as an extended polypeptide over the surface of calpains, contacting specific hydrophobic areas of the enzyme (Figure 6A). The α -helix from the N-terminal side of calpastatin passes through the calpain’s PEF Ca^2+^ binding domain, establishing a hydrophobic bond with the F610 residue (F597 in CANP2, Figure 6B). This contact stabilises the interaction between calpain and calpastatin and computational prediction of the protein-protein affinity between the F597C mutant and the inhibitor estimates a significant reduction in the stability of the resulting complex (∆∆G = −1.43 kcal/mol). Although the amino acid G492 (G479 in CANP2) is not in direct contact with calpastatin in this model, *in silico* analysis also predicts a reduction in the affinity between the inhibitor and the mutant calpain (∆∆G = −0.37 kcal/mol), possibly due to changes in the overall stability of the beta sheet domain in which G492 is located (Figure 1F). Altogether, the p. G492R/p. F610C compound heterozygous mutation may lead to a reduction in the affinity of the calpain inhibitor calpastatin for *CAPN1*, leading to increased calpain activity in patients and supporting the hypothesis of using calpain inhibitors as a therapeutic strategy in SMA (de la Fuente et al., 2019).

Our experiments using patient fibroblasts have not provided definitive information regarding which cellular and molecular pathways may be affected by the SMA4 causative *CAPN1* mutation. Autophagic processes, mitochondrial function and apoptotic pathways appeared unaltered in the SMA4 cells. Additionally, protein levels and intracellular distribution of β-catenin, a target of the protease activity of calpain-1 suggested as a molecular crosstalk between SMN and non-5q SMAs (Soltic and Fuller, 2020) that establishes functional association with a number of SMA genes (Figure 5B), remain normal in the SMA4 derived fibroblasts. Patient fibroblasts have proven to be a suitable model to investigate pathomechanisms in neurodegenerative diseases (Auburger et al., 2012), however the precise pathways activated by genetic mutations can be overlooked when not using complementary approaches, including spinal cord motor neurons and *in vivo* models (Juneja et al., 2019). In SMA, this has been recently illustrated by the vastly different autophagy profile between muscle, motor neurons and skin fibroblasts from a SMA patient (Sansa et al., 2021b). Further studies using neuronal *in vitro* and *in vivo* models bearing the p. G492R/p. F610C compound heterozygous mutation will allow describing the precise pathomechanisms leading to motor neuron loss in SMA. Our research provides another example of neurodegenerative diseases caused by mutations in calpain genes and increases the genetic heterogeneity of non-5q SMAs, adding *CAPN1* to the list of spinal muscular atrophy causative genes.

## Data Availability

The original contributions presented in the study are included in the article/Supplementary Material, further inquiries can be directed to the corresponding authors.
