# Supplementary material for: A Compound Heterozygous Mutation in Calpain 1 Identifies a New Genetic Cause for Spinal Muscular Atrophy Type 4 (SMA4)
Source: Front Genet. 2022 Jan 19;12:801253. doi: 10.3389/fgene.2021.801253 (PMC8807693; doi:10.3389/fgene.2021.801253)
Supplement: Supplementary file 1 [file DataSheet1.PDF]

Supplementary Table 1

Movement	Sh.Ab	ShAd	ElbE	ElbF	HipF	HipAd	HipE	KnE	KnF	ADf
2011	4	4	3	5	4	5-	5	3	4	5-
2021	4	4	2	4	3	3+	4+	2	3+	4

Supplementary Table 1. MRC scores for male proband. MRC scores are provided for indicated movements in 2011 and 2021 for weak groups only, wrists fingers plantar flexion and toes were and remained normal. All scores were symmetrical. ShAb/Ad = shoulder abduction/adduction. Elb. E, F = elbow extension, flexion. Kn = knee, ADf = ankle dorsiflexion

Supplementary Table 2

	Insertional	Spontaneous Activity			Volitional MUAPs					Max Volitional Activity		
Muscle	Insertional	Fibs	+ Wave	Fasc	Duration	Amplitude	Poly	Config	Recruitment	Amplitude	Pattern	Effort
Tibialis anterior.L	Normal	None	2+	2+	Normal	Sl. Incr.	1+	Normal	Normal	Normal	Reduced	Max.
Rectus femoris.L	Normal	2+	None	2+	Gr. Incr.	Sl. Incr.	2+	Normal	Early	Normal	Reduced	Max.
Vastus lateralis.R	CRD	2+	None	2+	Gr. Incr.	Sl. Incr.	2+	Normal	Early	Normal	Reduced	Max.
Deltoid.L	CRD	2+	1+	2+	Gr. Incr.	Sl. Incr.	1+	Normal	Early	Normal	Reduced	Max.

Supplementary Table 2. EMG scoring of sampled muscles for male proband. CRD = complex repetitive discharges. Fibs = fibrillation potentials. +Wave = positive sharp waves. Fasc=fasciculations. Poly = (excessively) polyphasic units. Sl / Gr Incr = slightly / greatly increased.

Supplementary Table 3: Multiple *in silico* analyses support the pathogenicity of the CAPN1 p.G492R and p.F610C variants.

Method	CAPN1 c.1474G>A (p.G492R)		CAPN1 c.1829T>G (p.F610C)	
	Score	Interpretation	Score	Interpretation
dbSNP153	rs17883283	Reported	rs200876514	Reported
gnomAD Exomes	247/248,888	Rare (0.0992%)	84/248,440	Rare (0.0338%)
GERP	5.26	Conserved	4.8699	Conserved
PhastCons100way	1	Conserved	1	Conserved
PhyloP100way	9.760	Conserved	7.669	Conserved
PolyPhen	1	Probably Damaging	1	Probably Damaging
PROVEAN	-6.88	Deleterious	-7.09	Deleterious
SIFT	0.000	Damaging	0.000	Damaging
Mutation Assessor	4.01	High impact	4.25	High impact
MutationTaster2	1	Disease Causing	1	Disease Causing
