# Supplementary material for: A Compound Heterozygous Mutation in Calpain 1 Identifies a New Genetic Cause for Spinal Muscular Atrophy Type 4 (SMA4)
Source: Front Genet. 2022 Jan 19;12:801253. doi: 10.3389/fgene.2021.801253 (PMC8807693; doi:10.3389/fgene.2021.801253)
Supplement: Supplementary file 2 [file DataSheet2.docx]

| Method | CAPN1 c.1474G>A (p.G492R) | | CAPN1 c.1829T>G (p.F610C) | |
| --- | --- | --- | --- | --- |
|  | Score | Interpretation | Score | Interpretation |
| dbSNP153 | rs17883283 | Reported | rs200876514 | Reported |
| gnomAD Exomes | 247/248,888 | Rare (0.0992%) | 84/248,440 | Rare (0.0338%) |
| GERP | 5.26 | Conserved | 4.8699 | Conserved |
| PhastCons100way | 1 | Conserved | 1 | Conserved |
| PhyloP100way | 9.760 | Conserved | 7.669 | Conserved |
| PolyPhen | 1 | Probably Damaging | 1 | Probably Damaging |
| PROVEAN | -6.88 | Deleterious | -7.09 | Deleterious |
| SIFT | 0.000 | Damaging | 0.000 | Damaging |
| Mutation Assessor | 4.01 | High impact | 4.25 | High impact |
| MutationTaster2 | 1 | Disease Causing | 1 | Disease Causing |

**Supplementary Table 1**

| Movement | Sh.Ab | ShAd | ElbE | ElbF | HipF | HipAd | HipE | KnE | KnF | ADf |
| --- | --- | --- | --- | --- | --- | --- | --- | --- | --- | --- |
| 2011 | 4 | 4 | 3 | 5 | 4 | 5- | 5 | 3 | 4 | 5- |
| 2021 | 4 | 4 | 2 | 4 | 3 | 3+ | 4+ | 2 | 3+ | 4 |

**Supplementary Table 1. MRC scores for male proband.** MRC scores are provided for indicated movements in 2011 and 2021 for weak groups only, wrists fingers plantar flexion and toes were and remained normal. All scores were symmetrical. ShAb/Ad = shoulder abduction/adduction. Elb. E, F = elbow extension, flexion. Kn = knee, ADf = ankle dorsiflexion

**Supplementary Table 2**

|  | **Insertional** | **Spontaneous Activity** | | | **Volitional MUAPs** | | | | | **Max Volitional Activity** | | |
| --- | --- | --- | --- | --- | --- | --- | --- | --- | --- | --- | --- | --- |
| Muscle | Insertional | Fibs | + Wave | Fasc | Duration | Amplitude | Poly | Config | Recruitment | Amplitude | Pattern | Effort |
| Tibialis anterior.L | Normal | None | 2+ | 2+ | Normal | Sl. Incr. | 1+ | Normal | Normal | Normal | Reduced | Max. |
| Rectus femoris.L | Normal | 2+ | None | 2+ | Gr. Incr. | Sl. Incr. | 2+ | Normal | Early | Normal | Reduced | Max. |
| Vastus lateralis.R | CRD | 2+ | None | 2+ | Gr. Incr. | Sl. Incr. | 2+ | Normal | Early | Normal | Reduced | Max. |
| Deltoid.L | CRD | 2+ | 1+ | 2+ | Gr. Incr. | Sl. Incr. | 1+ | Normal | Early | Normal | Reduced | Max. |

**Supplementary Table 2.** **EMG scoring of sampled muscles for male proband.** CRD = complex repetitive discharges. Fibs = fibrillation potentials. +Wave = positive sharp waves. Fasc=fasciculations. Poly = (excessively) polyphasic units. Sl / Gr Incr = slightly / greatly increased.

**Supplementary Table 3**: Multiple *in silico* analyses support the pathogenicity of the CAPN1 p.G492R and p.F610C variants.
